# Head injury in asylum seekers and refugees referred with psychological trauma

**DOI:** 10.1017/gmh.2016.23

**Published:** 2016-10-03

**Authors:** S. M. Doherty, R. Craig, M. Gardani, T. M. McMillan

**Affiliations:** 1 NHS Greater Glasgow and Clyde Psychological Trauma Service, Glasgow, UK; 2 Institute of Health and Wellbeing, MVLS, University of Glasgow, Glasgow, UK

**Keywords:** Comorbidity, head injuries, PTSD, refugees, trauma

## Abstract

**Objective.:**

Individuals who seek asylum are frequently fleeing violent persecution and may experience head injury (HI). However, little is known about the prevalence of HI in asylum seekers and refugees (ASR) despite the potential for HI to significantly affect cognitive and emotional functioning and to compromise asylum outcomes. This preliminary study investigates the prevalence of HI in ASR referred to a complex psychological trauma service.

**Method.:**

Participants were 115 adult ASR referred to a community psychological trauma service with moderate to severe mental health problems associated with psychological trauma. They were screened for a history of HI using a questionnaire developed for the study. Interpreters were used when required.

**Results.:**

The overall prevalence of HI was 51%. At least 38% of those with HI had a moderate–severe HI that could cause persisting disability. In 53% of those with HI, the cause was torture, human trafficking or domestic violence. Repeat HI can have cumulative effects on function; it was common, and was reported in 68% of those with HI. An injury to the head was not known to mental health clinicians prior to screening in 64% of cases.

**Conclusion.:**

The emotional and cognitive consequences of HI in ASR may increase the vulnerability of this disadvantaged group, and can be associated with neurobehavioural problems affecting daily life and may compromise asylum outcomes. Routine screening for HI in ASR is needed, as are links to neuropsychology and brain injury services for advice, assessment and intervention.

## Introduction

Asylum seekers and refugees (ASR) are by definition, more likely than the general population to have experienced physical assault and injury in their country of origin (United Nations, 2004), and to be victims of torture, including blows to the head and asphyxiation that can result in brain damage (Moreno & Grodin, 2002; Keatley *et al*. 2013). Mollica *et al*. (2014) reported that a high proportion (78%) of Vietnamese ex-political detainees resettled in Boston reported a history of head injury (HI). However, more generally there has been little empirical investigation into the prevalence of HI in the ASR population and as a consequence there is little informed guidance for service providers.

HI can result in long term impairments in attention, retrograde and anterograde memory, word finding and executive function (Cicerone *et al*. 2011). As a consequence, HI has the potential to affect the ability of an ASR to relate recent or historical information accurately or reliably and to understand and synthesise information needed to make decisions. Given this, and in light of the requirement for asylum applicants to provide a detailed account of their experiences, unidentified HI may increase the likelihood that an asylum applicant will provide an impoverished or unreliable account, be found ‘not credible’ and refused asylum. In addition to cognitive impairment, HI can cause emotional problems that can lead to the breakup of relationships, social isolation and unemployment that is associated with psychological distress and that can complicate effects of psychological trauma (McMillan *et al*. 2003). ASR often present to health services with complex needs including mental and physical health problems, social or housing problems and involvement in legal proceedings (Burnett & Peel, 2001) where cognitive impairment and emotional problems can be a disadvantage. Retrospective diagnosis of HI may be especially challenging in the case of ASR, where there may be no objective information about the occurrence or severity of the HI and where comorbidity and overlap between HI and mental health complaints (e.g. depression, post-traumatic stress disorder (PTSD)) is common (McMillan *et al*. 2003). Clinicians may not be alert to the possibility of HI when recording symptom complaints in a client group where there is already an expectation of a high prevalence of mental health problems including PTSD and depression (Fazel *et al*. 2005). A better understanding of the prevalence of HI in this population is potentially crucial if an adequate humanitarian and clinical response to the needs of this vulnerable group is to be provided.

## Methods

Participants were adult ASR referred to the NHS Greater Glasgow and Clyde Psychological Trauma Service in Scotland. This is a tertiary mental health service for ASR with moderate to severe complex mental health problems stemming from trauma, which receives referrals from general practitioners and other mental health services. Trauma experienced by clients referred into the service commonly includes detention and torture, rape, war, human trafficking, female genital mutilation, domestic violence and childhood abuse. Over a 12-month period, screening for HI was carried out routinely in clients being seen for their initial mental health assessment or for review using a questionnaire designed specifically for this purpose (see online Supplementary file 1) To be included, participants had to be ASR and aged 18 or over. Where English was not the first or preferred language, an interpreter was used. Severity of HI was coded according to service user reports of loss of consciousness (LoC), with durations of <30 min classified as mild and >30 min as moderate–severe (Holm *et al*. 2005).

## Results

### Participation and characteristics of the sample

During the 12-month period of study, 135 ASR were referred to or were already being seen by the service. Of these, screening for HI was not attempted in 20 as it was clinically contra-indicated because of distress, because they were unwilling or did not attend any appointments. HI screening was attempted in the remaining 115. There were 28 countries of origin, with 43% of participants from Africa, 32% from the Middle East, 12% from South Asia, 10% from North Asia and 3% from Eastern Europe. There were 68 females (59%) and 47 males (41%) with average age 36 (s.d. 9) years (range 18–71). In 59 (54%) participants an interpreter was required and 80 (75%) had at least some use of English. The median length of time since arriving in the UK was 4 years (inter quartile range 2.0, 6.5; *n* = 77). Information on history of HI was obtained in 103. HI and non-HI groups did not differ in age, need for an interpreter, years living in the UK or presence of a physical health problem. Females were more common in the non-HI group (see Table 1).

**Table 1.** Characteristics of service users with and without head injury *(*HI*)*

	HI (*n* = 53)	No HI (*n* = 50)	
Age	34.8 (s.d. 8.1); *n* = 50	38.0 (s.d. 10.9); *n* = 47	*t* = −1.66; *p* = 0.10
Gender			
Male	53% (*n* = 28)	28% (*n* = 13)	*χ*^2^ = 6.52; *p* = 0.011
Female	47% (*n* = 25)	72% (*n* = 34)	
Interpreter needed	50% (*n* = 26)	63% (*n* = 29)	*χ*^2^ = 1.69; *p* = 0.194
Years in UK	4.8 (s.d. 4.5); *n* = 34	5.2 (s.d. 3.6); *n* = 35	*t* = −0.384; *p* = 0.702
Physical health problem	56%; *n* = 24	43%; *n* = 17	*χ*^2^ = 2.70; *p* = 0.259

### Prevalence of HI

HI screening was completed in 103 participants, and in the remaining 12, only partly completed due to distress (*n* = 10) or moving away from Glasgow between appointments (*n* = 2). At least one HI with LoC was reported by 53 of 103 (51%) completing the screening. In the remainder, 47 (46%) reported no history of HI and three (3%) were unsure. A HI sustained in the UK was reported by 10/103.

### Characteristics of the HI sample (*N* = 53)

In relation to the most severe HI that each person reported (i.e. longest LoC duration); 17 (33%) reported an accidental cause (nine falls and eight road traffic accidents) and 34 (67%) assault. Of the assaults, 18 were related to torture, six to domestic violence, four to violence during human trafficking (in the UK), and six to assaults not-specified, which could have been any of the foregoing or other violent causes. Hence, a HI resulting from domestic violence or violence associated with torture or trafficking was reported in at least 53% (28/53) of those with a HI. Some could not say what age they were when their HI occurred; in 43 that did, the median age at injury for the most severe HI was 25 years (IQR; 15, 32; range 4–42). Median time since injury in those able to estimate, was 10 years (IQR 5, 13; range 0.5–38; *n* = 41).

The estimated maximum duration of LoC was <30 min in 11/53 (21%) suggesting ‘mild’ HI. In 20 (38%) moderate–severe HI with LoC >30 min was suggested (LoC 0.5–3 h in 11 and >3 h in 9); the remaining 22 (41%) participants were unsure of LoC duration. In terms of the overall sample, this suggests that in 20/103 (19%) a single HI of a severity that might be disabling was reported and with a further 21% being uncertain of LoC duration. In relation to the most serious HI sustained outwith the UK, 50 participants provided information; 26/50 (52%) reported attending hospital after HI, with 14 (28%) admitted for up to 72 h; 9 (18%) for 4–10 days, six (12%) for >10 days and two (4%) uncertain. None reported brain surgery. Of 41 providing information on the number of HI sustained, more than one HI was reported in 28 (68%), and of these 18/28 reported more than five HI.

Clinicians said they were not aware of any HI before screening in 34 of the 53 (64%) HI cases. Twenty-seven (51%) said that they were currently affected by the event in which their most severe HI occurred, 10 (19%) were unsure if it affected them currently, 11 (22%) said it did not and data were incomplete for 5 (9%).

### HIs sustained in the UK

Ten out of 51 (20%) participants said they sustained a HI in the UK and of these, four reported attending hospital. One reported contact with UK brain injury rehabilitation services. In five the cause of HI was accidental and in five a result of violence during trafficking for sexual exploitation. In two the HI in the UK was the only HI reported.

## Discussion

The prevalence of HI of a severity likely to cause persisting disability is estimated to be about 2% in the general population in Western countries (Langlois *et al*. 2004; Tagliaferri *et al*. 2006). In the present study, HI of a severity that is likely to result in persisting disability was estimated in at least 19% (*n* = 20) of the sample, with a further 21% unsure of their duration of LoC. This estimate is high relative to Western prevalence estimates. In broad terms it is consistent with the findings of a retrospective US study of 566 torture survivors seeking treatment for medical and psychiatric problems, where 15% reported HI with LoC for more than 30 min (Keatley *et al*. 2013). The estimate of HI with any LoC was 51% in the present study and as such is similar to the estimated prevalence of 54% in Vietnamese survivors of torture living in the USA who reported HI with LoC (Mollica *et al*. 2014). Torture was a common cause of HI in our sample, and while the neurological sequelae of torture are documented (Rasmussen, 1990; Moreno & Grodin, 2002), the link between torture and HI in ASR has received limited empirical attention (Mollica *et al*. 2014). Moreover, torture, domestic violence and human trafficking rarely feature as HI causes in epidemiological studies in Western countries, where road traffic accidents and falls predominate (Langlois *et al*. 2004; Tagliaferri *et al*. 2006). The link between human trafficking and HI in the UK also warrants further attention. We consider that the high proportion of female ASR in this study (59%) might reflect the policy of dispersing families as opposed to single persons to Glasgow during the study period because of the type of accommodation available in the city.

The high prevalence of HI reported by ASR in the current study is sobering, given that clinicians were often unaware of the HI. Several factors might explain the failure to spontaneously disclose a history of HI, including the long time since HI, avoidance of trauma reminders, cognitive impairment and participants – not realising that that their HI is potentially indicative of brain injury. In the West, hospitalised HI is mild in more than 90% of cases and a good outcome is expected in most (Tagliaferri *et al*. 2006), whereas 38% of head injured cases in the current study reported that their worst HI was moderate–severe, where persisting effects are likely (McMillan *et al*. 1996), particularly in the absence of hospital care which was common in this sample. Moreover, repeated HI, even if mild, can have a cumulative effect on cognitive and emotional functioning (Thornton *et al*. 2008) and the majority of HI participants reported more than one HI, and a significant minority, five or more. Contact with brain injury services was rare, even when HI had occurred in the UK and this would also be associated with poorer outcome (Cicerone *et al*. 2011).

Unpicking the role of brain injury from other potential causes of emotional disturbance in ASR is difficult as an association between brain injury and depression or PTSD might reflect a cumulative effect of exposure to psychological trauma rather than of brain injury *per se*. In a pilot study, Mollica *et al*. (2009) reported brain MRI in a sample of torture survivors from Vietnam who reported HI, and found reduced cortical thickness in prefrontal and temporal areas and reduced volume in the amygdala compared with controls with no self-reported history of brain injury. A higher occurrence of depression was associated with thinning in some prefrontal and temporal areas. However, given that imaging studies report abnormalities of brain structure in people exposed to psychological trauma, and in particular reduced volume in the amygdala, findings of this kind are difficult to interpret with confidence (O'Doherty *et al*. 2015). The recent work by Mollica and collaborators indicates a high prevalence of depression and PTSD in torture survivors with HI (Mollica *et al*. 2014). These findings by Mollica *et al*. (2014) if taken together with the findings from the current study highlight the importance of screening for HI in ASR attending mental health services.

### Limitations

Findings from the current study relate to individuals who had previously been identified as suffering from moderate–severe mental health problems and they may not therefore represent the ASR population more generally. Clinicians could not attempt or complete screening with all service users, usually because of distress linked to psychological trauma and inevitably there is missing data, although this is unlikely to alter the key messages in the study. The screening questionnaire relies on self-report and estimation of LoC to indicate the presence and severity of HI and this may be inaccurate; some participants reported little or no memory of events and did not know if they had sustained LoC. Other retrospective assessments of HI severity such as Post-Traumatic Amnesia (McMillan *et al*. 1996) are difficult to use in a complex trauma group because retrospective assessment of PTA requires systematic prompting about memories for events around the time of the injury and this might have potential to re-traumatise this client group. Future studies should explore cognitive impairment and disability in order to better understand the potential impact of HI. These assessments need to take into account cultural and language differences that may affect test validity and their interpretation and potential impairment arising from mental health disorders as well as from HI.

### Service implications

The asylum process relies on applicants providing a detailed, chronological account of experiences leading to seeking asylum. Case ‘credibility’ is judged partly on the degree to which memory for specific events and country of origin information is coherent and consistent. Where applicants have poor recall, they may be viewed as ‘not credible’. While the link between traumatic stress and fragmented recall in asylum seekers is known (Herlihy & Turner, 2007), the present study raises the possibility that in some cases, cognitive impairment after HI might be a factor in poor recall. The emotional effects of HI include impulsivity, impaired judgement and aggression and may lead to neurobehavioural difficulties that in turn can be associated with social and employment problems and involvement with the criminal justice system (Wood & McMillan, 2001). Given that HI may have significant implications for the individual trying to rebuild their life after escaping the trauma of persecution, there is a strong argument for incorporating screening for HI into routine health assessments. This is reinforced by our finding that mental health clinicians were often unaware of a history of HI in their clients prior to screening. This study also highlights the need for mental health professionals working with ASR to consider HI as a potential factor underpinning psychological distress and poor functioning and in this event to consider adjusting psychological interventions to take account of cognitive impairment. Staff education and training will be important to achieve this. Clinical judgment is needed when deciding if or when assessment for HI is appropriate given the potential for questioning to evoke traumatic memories. Closer links between brain injury and mental health services for ASR is also needed to ensure that this disadvantaged population has access to clinical neuropsychology and brain injury services.

### Conclusion

HI is a potentially important contributor to poor functioning in ASR presenting to mental health services. Our preliminary findings suggest that screening for HI in the ASR population is important and that there is a need to improve links between mental health and brain injury services.

## Supplementary material

For supplementary material accompanying this paper visit http://dx.doi.org/10.1017/gmh.2016.23. click here to view supplementary material
