# Supplementary material for: Head injury in asylum seekers and refugees referred with psychological trauma
Source: Glob Ment Health (Camb). 2016 Oct 3;3:e28. doi: 10.1017/gmh.2016.23 (PMC5454765; doi:10.1017/gmh.2016.23)
Supplement: Supplementary file 1 [file S2054425116000236sup001.docx]

**Head Injury Screening Form ( V5a)**

Client Name: ___________________________

Date of Birth: ___________________________

Gender: **M / F**

Date of assessment: ______________________

Country of origin: _________________________

**Client Study Number - _____**

Language Spoken: ________________________

Interpreter required? **Y / N**

Any English Language? ___________________

Length of time in UK: ______________________

Referral Source __________________________

Any Physical health problems? _______________ Total CORE Score _______________________

________________________________________ Clinician completing ______________________

**🞎 It was not possible / appropriate to complete screening questionnaire - Please give details: ______________________________________________________________________________**

1. **Have you ever had an injury causing you to be “knocked out”? For example, being hit on the head or being involved in a car accident.** - **Yes / No**

(*If yes, continue with further questions*)

1. **How many times has this happened?** (If there are multiple events an approximate number is enough).

_______________*(If more than 1 – may need to complete full screening questionnaire)*

1. **What was the longest time you have been knocked out for?** _______________

How do you know this? (Did someone tell you? Is it from the gap in your memory?)

1. **When did this injury happen?** _______________________
2. **What country did the injury take place in?** ______________________________
3. **What was the cause of the injury?**
4. **Did you go to hospital?** - **Yes / No**

If yes, how long did you stay in hospital? Did you have an operation to your brain?

___________________________ _____________________________

1. **Have you had any contact with brain injury services in the UK?** - **Yes/ No**

(*If yes, ask for details*):

1. **Do you think the situation that caused injury to your head affects you now? If so, how?**

*(If necessary, prompt with the following examples):*

For example, since the event that caused injury to your head, have you noticed any of these symptoms?

Headache Poor sleep

Memory problems Fits

Dizziness Irritability

Problems concentrating Anxiety

Fatigue Low mood/Depression

1. **Is there anything else related to these experiences that you think is important that we haven’t asked about?**
2. **For Clinician –**

Were you already aware of the event in which the head injury was sustained? **- Yes/ No**

Was this event the reason the client was referred to the service?**- Yes/ No**

**If client reports more than one head injury:**

1. **Have you ever had an injury causing you to be “knocked out” since arriving in the UK?** - **Y / N**

For example, being hit on the head or being involved in a car accident.

*(f yes, complete further questions. If no, questionnaire is complete.)*

1. **When did this injury happen?** _______________________
2. **What was the cause of the injury?**
3. **Did you go to hospital?** - **Yes/ No**

If yes, how long did you stay in hospital? Did you have an operation to your brain?

__________________________ ____________________________

1. **Have you had any contact with brain injury services in the UK as a result of this injury?**- **Y /N**

(*If yes, ask for details*)

1. **Do you think the situation that caused injury to your head affects you now? If so, how?**

*(If necessary, prompt with the following examples):*

For example, since the event that caused injury to your head, have you noticed any of these symptoms?

Headache Poor sleep

Memory problems Fits

Dizziness Irritability

Problems concentrating Anxiety

Fatigue Low mood/Depression
